# Renal Epithelial Complement C3 Expression Affects Kidney Fibrosis Progression

**DOI:** 10.3390/ijms252312551

**Published:** 2024-11-22

**Authors:** Ganna Stepanova, Anna Manzéger, Miklós M. Mózes, Gábor Kökény

**Affiliations:** 1 Institute of Translational Medicine, Semmelweis University, Nagyvárad tér 4, 1089 Budapest, Hungary; gannastepanova2016@gmail.com (G.S.); manzeger.anna@semmelweis.hu (A.M.); mozesmiklos@yahoo.com (M.M.M.); 2 International Nephrology Research and Training Center, Semmelweis University, Nagyvárad tér 4, 1089 Budapest, Hungary

**Keywords:** tubulointerstitial fibrosis, progression, complement, gene expression, FSGS

## Abstract

Kidney fibrosis is a hallmark of chronic kidney diseases. Evidence shows that genetic variability and complement component 3 (C3) might influence tubulointerstitial fibrosis. Still, the role of renal C3 production in the epithelial-to-mesenchymal transition (EMT) and genetically determined fibrosis progression remains undiscovered. The kidneys of fibrosis-resistant C57Bl/6J (B6) and fibrosis-prone CBA/J (CBA) and BALB/cJ (BalbC) mice (n = 4–8/group) were subjected to unilateral ureteral obstruction (UUO) and analyzed after 1, 3, and 7 days, along with human focal glomerular sclerotic (FSGS) and healthy kidneys. Mouse primary tubular epithelial cells (PTECs) were investigated after 24 h of treatment with transforming growth factor β (TGFβ) or complement anaphylatoxin 3a (C3a) agonist (n = 4/group). UUO resulted in delayed kidney injury in fibrosis-resistant B6 mice, but very early renal C3 messenger RNA (mRNA) induction in fibrosis-prone CBA and BalbC mice, along with collagen I (Col1a1) and collagen III (Col3a1). CBA depicted the fastest fibrosis progression with the highest C3, lipocalin-2 (Lcn2), Tgfb1, and chemokine (C-C motif) ligand 2 (Ccl2) expression. Human FSGS kidneys depicted C3 mRNA over-expression and strong tubular C3 immunostaining. In PTECs, C3a agonist treatment induced pro-fibrotic early growth response protein 1 (EGR1) expression and the EMT, independent of TGFβ signaling. We conclude that de novo renal tubular C3 synthesis is associated with the genetically determined kidney fibrosis progression rate in mice and the pathogenesis of FSGS in humans. This tubular C3 overproduction can, through local pro-fibrotic effects, influence the progression of chronic kidney disease.

## 1. Introduction

Chronic kidney disease (CKD) is an increasing healthcare problem. Over 15% of adults in the United States suffered from CKD in 2019, and an estimated 5–10 million deaths are reported annually worldwide. Additionally, the incidence of CKD is continuously rising [1]. CKD occurs due to various diseases, including diabetes, hypertension, and glomerulonephritis [2], leading to end-stage renal disease (ESRD) and, consequently, lifetime dialysis or kidney transplantation. Independent of its cause, the final common pathway for CKD is renal fibrosis, a poorly understood process [3,4] that lacks efficient drugs to repair damage, making kidney fibrosis a vital research subject [5].

Renal fibrosis is characterized by glomerular sclerosis, tubular injury, atrophy, tubular-interstitial fibrosis, and proteinuria [6]. Furthermore, understanding this disease is complicated by the varying progression rates among patients, even those with the same initial disease [3], such as diabetic and hypertensive nephropathies [7]. We previously demonstrated in rodent models that genetic background influences individual disease progression rates [8,9] and that both early growth response protein 2 (EGR2) and tissue metallopeptidase inhibitor-1 (TIMP-1) play vital roles in its pathogenesis [10,11,12]. The epithelial-to-mesenchymal transition (EMT) is considered to be an essential process in fibrosis. Myofibroblasts emerge as key players in tubulointerstitial damage and fibrosis, leading to the increased synthesis of alpha-smooth muscle actin (α-SMA or ACTA2) and extracellular matrix (ECM) components (type I, type III collagens, andfibronectin) and enabling mononuclear inflammatory cell infiltration [13]. TGF-β is one of the most potent inducers of fibrotic processes, boosting further ECM production and indirectly inhibiting matrix degradation via TIMP-1 overproduction. Still, the genetic basis for variation in the progression of renal fibrosis remains poorly understood.

Recent studies suggest that the complement system might play a pivotal role in the progression of CKD and tubulointerstitial fibrosis [14,15,16,17]. An essential aspect of the innate immune system is that the renal complement system comprises circulating and locally synthesized factors that recognize pathogens, immune complexes, and cell debris [18,19]. Research has demonstrated a significant role for complement activation along the classical and alternative pathways in CKD progression [17,18]. C3a is the cleavage product of C3 and acts as an anaphylatoxin that can exert its effects when binding to its C3a receptor (C3aR). C3aR is expressed in human and murine renal tubular epithelial cells [20]. Moreover, the activation of C3aR in human primary tubular epithelial cells treated with C3a resulted in a rapid and significant type-1 pro-collagen gene and protein expression [20], implicating the involvement of C3a in tubulointerstitial fibrosis.

Furthermore, renal C3 depositions were associated with the progression of proteinuric nephropathy in vitro and in vivo [21]. The elimination of the C3a receptor reduced the severity of tubulointerstitial fibrosis, and the inhibition of C3 likewise reduced renal inflammation and fibrosis and suppressed EMT [22,23,24]. Interestingly, C3 was described as a significant risk factor for human immunoglobulin A (IgA) nephropathy progression [25]. Previous studies revealed that the sera of FSGS patients contain elevated levels of C3a [26,27]. Han and colleagues reported that treating the human proximal tubular cell line HK-2 with the sera of FSGS patients exerted a pro-fibrotic response with induced fibronectin and type I collagen expression that was effectively blocked by a C3aR antagonist [26]. They observed a similar effect when treating HK-2 cells with native human C3a. Additionally, several renal cell types, including tubular epithelial cells, can produce complement components, including C3 [28,29,30,31]. Although evidence has shown the pathogenic effect of C3a/C3aR in chronic tubulointerstitial diseases, its direct impact on the kidney and its exact molecular mechanism remain obscure. It is also unclear whether genetic differences affect local renal C3 production.

In this study, we aimed to elucidate the impact of the intra-renal complement C3 in renal fibrosis by using both in vivo and in vitro research models. We also aimed to investigate whether the genetically determined kidney fibrosis progression rate in mice is related to renal C3 mRNA and gene expression differences. We further wanted to test the expression of inflammatory and fibrotic markers, as well as pro-inflammatory and pro-fibrotic transcription factors, which could potentially lead to therapeutic development. In addition, we aimed to elucidate the molecular pathways associated with the progression of kidney fibrosis by subjecting mouse kidney epithelial cells to a C3a agonist. Finally, in human FSGS kidney biopsy samples, we aimed to observe the localization of C3.

## 2. Results

### 2.1. Renal Histomorphology Depicts Delayed Fibrotic Response upon UUO in B6 Kidneys

**Figure 1:**
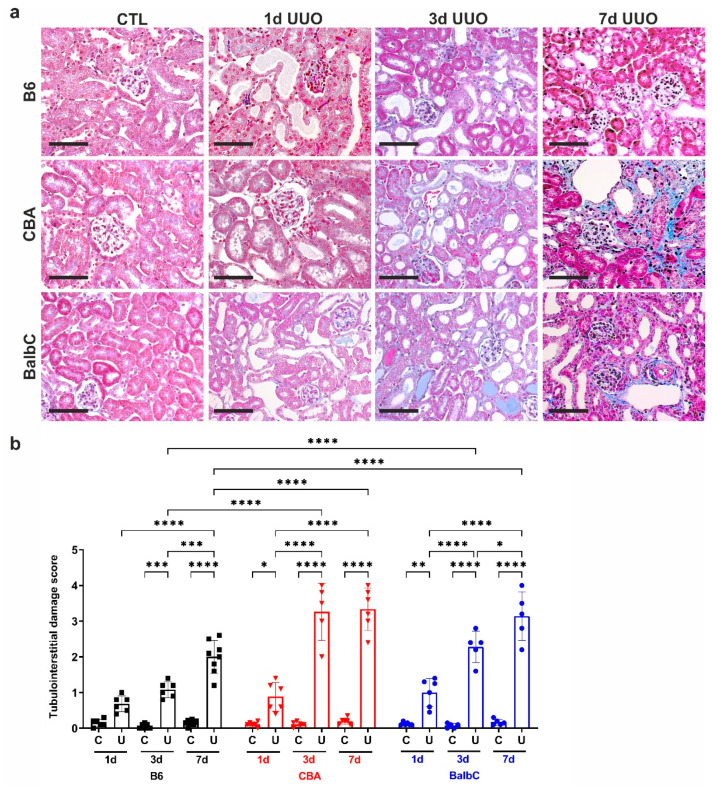
Renal histology with (**a**) representative photomicrographs of B6, CBA, and BalbC contralateral (CTL) and obstructed kidneys (UUO) and (**b**) evaluation of tubulointerstitial damage scores at 1/3/7 days (1d, 3d, 7d) after surgery in CTL (C) and UUO (U) kidneys. Masson’s trichrome stain, magnification 400×. The scale bar represents 50 μm. Two-way ANOVA and Holm–Sidak’s multiple comparison test (n = 5–8/group; * *p* < 0.05, ** *p* < 0.01, *** *p* < 0.001, and **** *p* < 0.0001).

The trichrome staining of control contralateral (CTL) and obstructed (UUO) kidneys 24 h after surgery revealed no substantial damage at this early time point. Only a slight tubular dilation was present in all obstructed kidneys, regardless of mouse strain, without a detectable fibrotic response or inflammatory cell infiltration (Figure 1, UUO 1d vs. CTL).

Strain differences appeared on Day 3 after UUO surgery, as CBA and BalbC UUO kidneys showed progression of the tubular dilations and the initiation of interstitial ECM deposition (Figure 1a,b). A significant percentage of tubules in the CBA and BalbC UUO kidneys depicted epithelial atrophy and tubular hyaline accumulation on Day 3 as compared to B6 UUO kidneys (Figure 1b). In BalbC kidneys, the pathological changes progressed to Day 7, with a significant degree of interstitial fibrosis (Figure 1a,b, UUO 7d) and more intense tubular atrophy with dilations. In contrast, the B6 UUO kidneys depicted only prominent tubular dilations with mild atrophy on Day 3 and mild fibrosis along with tubular damage on Day 7 (Figure 1a,b).

**Figure 2:**
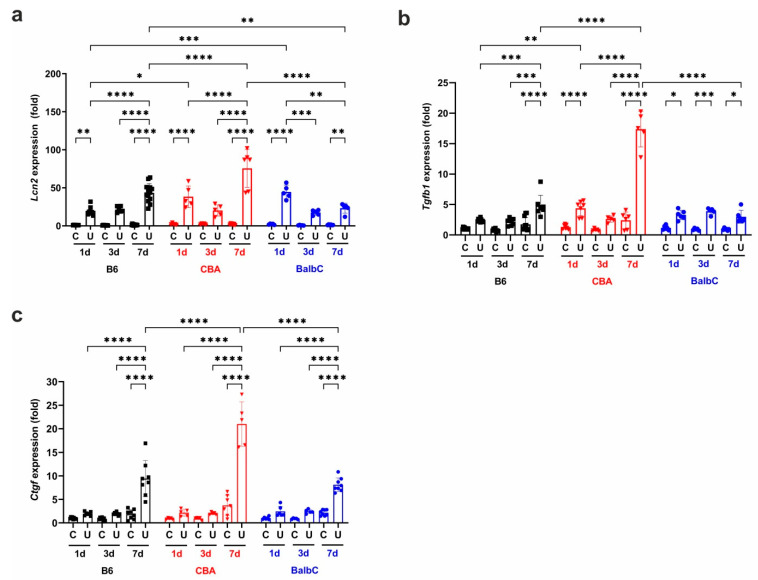
Renal Lcn2, TGFβ, and CTGF mRNA expressions at 1/3/7 days after UUO. Relative fold change mRNA expressions of (**a**) lipocalin-2 (*Lcn2*), (**b**) TGFβ (*Tgfb1*), and (**c**) CTGF (*Ctgf*) in control contralateral (C) and obstructed (U) kidneys of B6, CBA, and BalbC mice were calculated against *18S* rRNA expression. Data were analyzed via two-way ANOVA and Holm–Sidak’s post-hoc test (* *p* < 0.05, ** *p* < 0.01, *** *p* < 0.001, and **** *p* < 0.0001). n = 5–13/group.

Per histology, the mRNA expression of *lipocalin-2* or NGAL (*Lcn2*), a marker of tubular damage, showed dysregulation on Day 1 and Day 7 between all three strains of mice, with a significantly lower expression in the BalbC strain on Day 7. The CBA strain depicted the strongest tubular damage on Day 7. Compared to CBA, B6 kidneys had the mildest *Lcn2* over-expression on Day 1 and Day 7 (Figure 2a).

### 2.2. Gene Expressions of Fibrosis Markers Were Upregulated in All Strains Early After UUO

In the CBA and BalbC strains, UUO induced an immediate renal expression of pro-fibrotic *Tgfb1* mRNA on Day 1 (Figure 2b). We observed dysregulation between the strains on Day 1 and 7, with CBA showing the highest over-expression. Interestingly, BalbC kidneys exhibited a delayed progression in *Tgfb1* expression from Day 1 to Day 7, similar to B6. Additionally, connective tissue growth factor (*Ctgf)* expression also depicted a slow progressive increase from Day 1 to Day 7 after UUO in all strains, depicting the most robust increase in CBA by Day 7 (Figure 2c).

**Figure 3:**
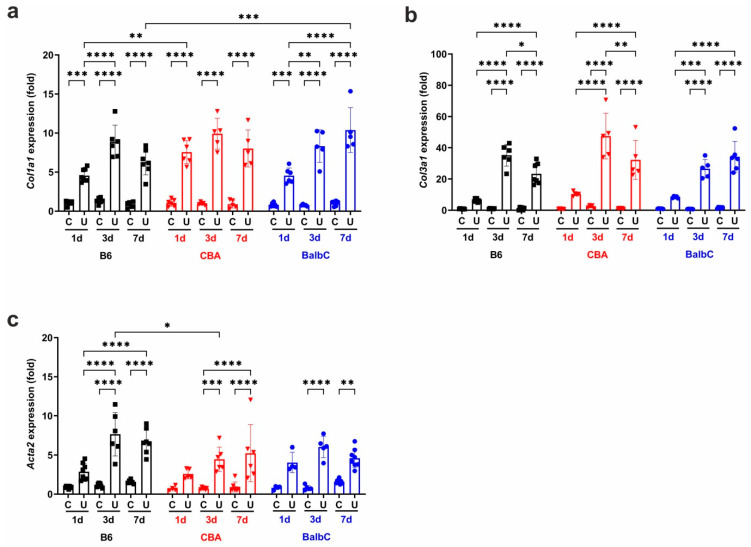
Renal mRNA expression of type I and type III collagens and α-SMA at 1/3/7 days after UUO. Relative fold change mRNA expressions of (**a**) type I collagen (*Col1a1*) and (**b**) type III collagen (*Col3a1*) and (**c**) α-SMA (*Acta2*) in control contralateral (C) and obstructed (U) kidneys of B6, CBA, and BalbC mice were calculated against *18S* rRNA expression. Data were analyzed via two-way ANOVA and Holm–Sidak’s multiple comparison test (* *p* < 0.05, ** *p* < 0.01, *** *p* < 0.001, and **** *p* < 0.0001; n = 4–8/group).

The gene expression patterns of collagens (*Col1a1* and *Col3a1*) followed *Ctgf* and were upregulated in all UUO kidneys on Day 1, with CBA showing the highest increase (Figure 3a,b). The collagen expressions in UUO kidneys progressed until Day 3, when strain-dependent differences vanished, and the expression levels remained comparable until Day 7 (Figure 3a,b).

During renal EMT, smooth-muscle actin (α-SMA or *Acta2*) is expressed in tubular epithelial cells. In our study, *Acta2* gene expression was triggered by UUO in all mouse strains from Day 1 (although it did not reach statistical significance), increased progressively to Day 3, and remained high until Day 7 in all strains (Figure 3c).

**Figure 4:**
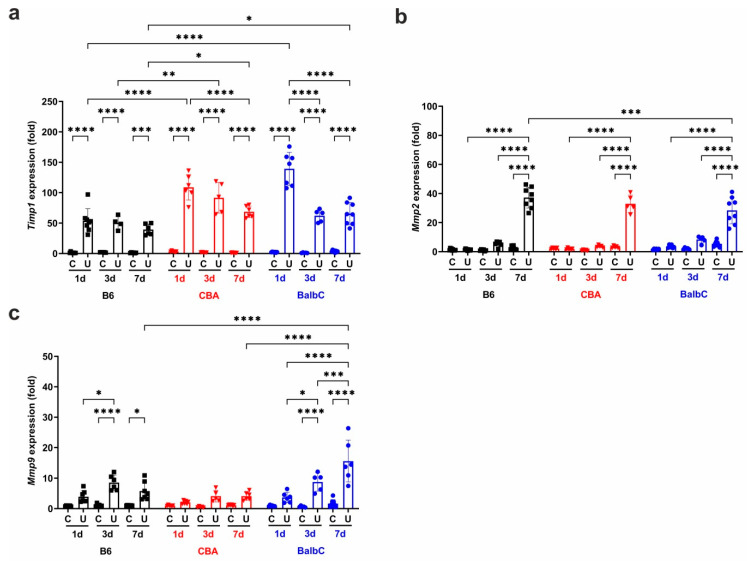
Renal mRNA expression of TIMP-1, MMP2, and MMP9 at 1/3/7 days after UUO. Relative fold change mRNA expressions of (**a**) tissue inhibitor of metalloprotease-1 (*Timp1*), (**b**) matrix metalloprotease 2 (*Mmp2*), and (**c**) matrix metalloprotease 9 (*Mmp9*) in control contralateral (C) and obstructed (U) kidneys of B6, CBA, and BalbC mice were calculated against 18S rRNA expression. Data were analyzed via two-way ANOVA and Holm–Sidak’s multiple comparison test (* *p* < 0.05, ** *p* < 0.01, *** *p* < 0.001, and **** *p* < 0.0001; n = 4–8/group).

UUO induced an immediate dysregulation of *Timp1* in all strains, with a significantly higher expression in CBA and BalbC kidneys than in B6 ones (Figure 4a), and this difference remained constant until Day 7. The expression of ECM-degrading matrix metalloproteinase-2 (*Mmp2)* was slightly but not significantly upregulated by UUO on Day 1 and Day 3 in all strains. Still, a time-dependent progressive increase could be observed (Figure 4b), reaching a comparable level in UUO kidneys of all strains by Day 7. The expression of matrix metalloproteinase-9 (*Mmp9)* progressively increased in both B6 and BalbC kidneys (but not in CBA ones) from Day 1 to Day 3 after UUO (Figure 4c), but on Day 7, increased further only in BalbC UUO kidneys.

### 2.3. UUO Induced Early Over-Production of Pro-Fibrotic Transcription Factors Dominantly in BalbC and CBA Kidneys

**Figure 5:**
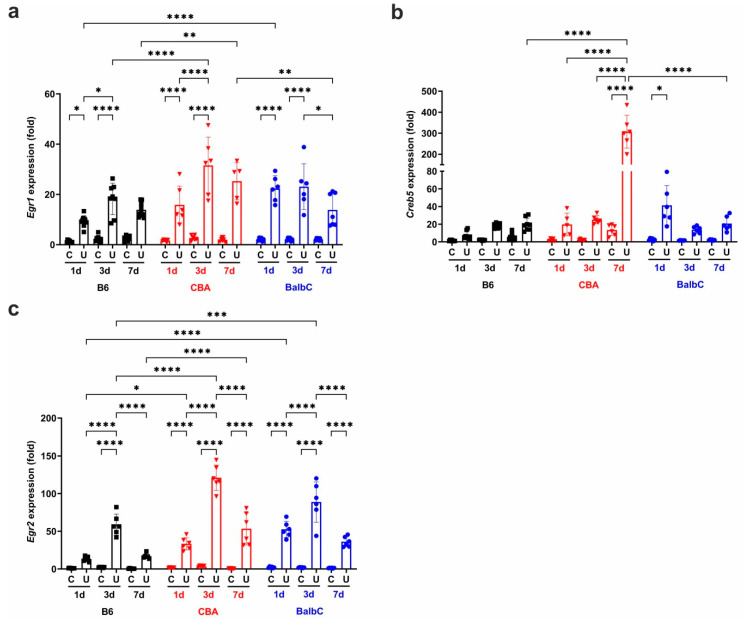
Renal mRNA expression of transcription factors EGR1, CREB5, and EGR2 at 1/3/7 days after UUO. Relative fold change mRNA expressions of (**a**) EGR1 (*Egr1*), (**b**) CREB5 (*Creb5*), and (**c**) EGR2 (*Egr2*) in control contralateral (C) and obstructed (U) kidneys of B6, CBA, and BalbC mice were calculated against *18S* rRNA expression. Data were analyzed via two-way ANOVA and Holm–Sidak’s multiple comparison test (* *p* < 0.05, ** *p* < 0.01, *** *p* < 0.001, and **** *p* < 0.0001; n = 4–8/group).

UUO induced immediate and robust *Egr1* mRNA expression in CBA and BalbC UUO kidneys, and to a lesser extent in B6 kidneys, on Day 1. This discrepancy between B6 and CBA increased further by Day 3, and the *Egr1* expression on Day 7 increased further in CBA, but dropped in BalbC to similar levels as those in B6 kidneys (Figure 5a).

On Day 1 post-UUO, the expression of cyclic AMP-responsive element-binding protein 5 (*Creb5)* was strongly induced in the BalbC strain, but also tended to increase in CBA. On Day 3, all expressions of *Creb5* equalized in UUO kidneys (Figure 5b). Interestingly, on Day 7, we observed a dramatic *Creb5* over-expression in CBA UUO kidneys, whereas in B6 and BalbC kidneys, there were no significant differences between the obstructed and control kidneys.

The expression of *Egr2* (Figure 5c) also had a robust initiation in the CBA and BalbC strains. By Day 3, the *Egr2* expressions in all UUO kidneys were elevated further, with a significantly milder increase in B6. On Day 7, *Egr2* expression was slightly reduced, with that of CBA remaining the highest.

### 2.4. Early STAT3 Over-Activity Remained Elevated for Seven Days After UUO

**Figure 6:**
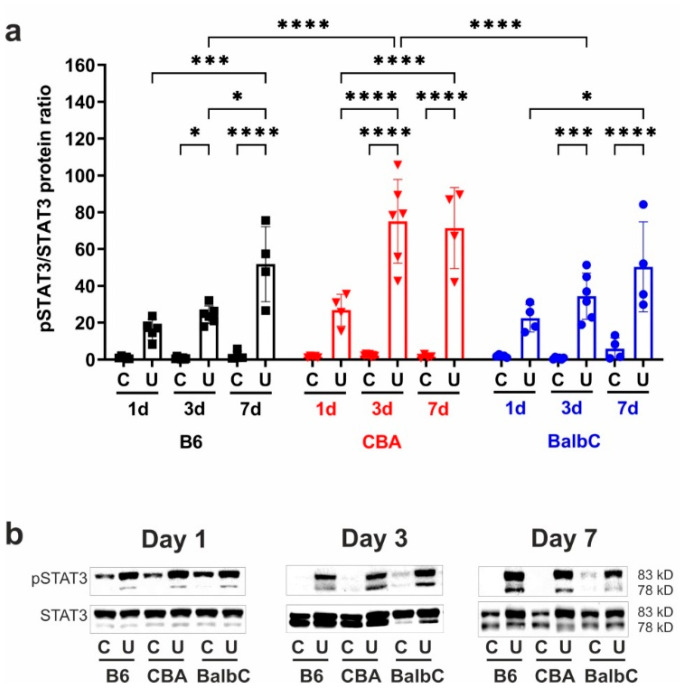
Phosphorylation of STAT3 in kidneys at 1/3/7 days after UUO. The ratio of phosphorylated STAT3 (pSTAT3, at Tyr705) and total STAT3 (STAT3) in control contralateral (C) and obstructed (U) kidneys of B6, CBA, and BalbC mice were normalized to the calibrator sample (**a**); representative blots are shown in panel (**b**). Data were analyzed via two-way ANOVA and Holm–Sidak’s multiple comparison test (* *p* < 0.05, *** *p* < 0.001, and **** *p* < 0.0001; n = 4–6/group).

The activation of signal transducer and activator of transcription 3 (STAT3) was observed as an increase, although without statistically significant phosphorylation (at Tyrosine 705) 24 h after UUO in all strains (Figure 6). STAT3 phosphorylation increased progressively in all strains, showing the highest activity in CBA UUO kidneys on Day 3 compared to B6 and BalbC kidneys. By Day 7, STAT3 phosphorylation activity reached equally high levels in the UUO kidneys of all strains (Figure 6).

### 2.5. UUO-Induced Strain and Time-Dependent Renal Complement Expression Pattern

**Figure 7:**
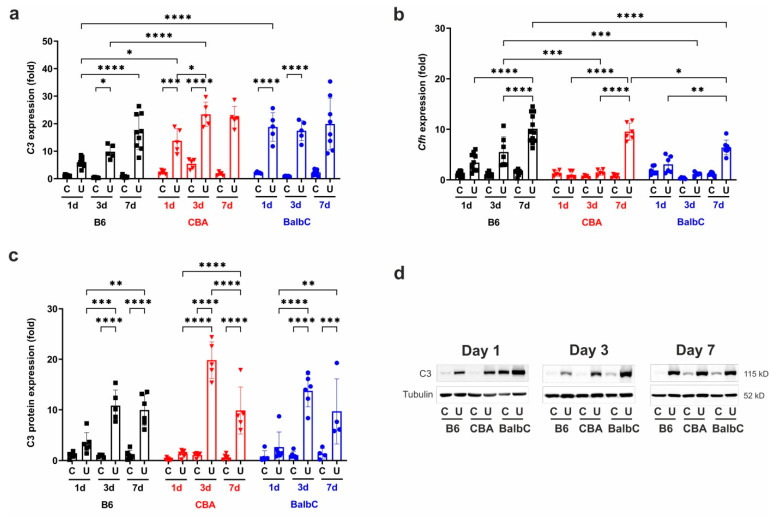
Strain- and time-dependent renal C3 and CfH expressions after UUO. Relative fold change mRNA expressions of (**a**) complement C3 (*C3*), (**b**) complement Factor H (*Cfh*), and (**c**) protein expression of complement C3 (C3) in control contralateral (C) and obstructed (U) kidneys of B6, CBA, and BalbC mice were calculated against *18S* rRNA and tubulin expression, respectively. Representative blots from Day 1, 3 and 7 kidneys are shown in panel (**d**). Data were analyzed via two-way ANOVA and Holm–Sidak’s multiple comparison test (* *p* < 0.05, ** *p* < 0.01, *** *p* < 0.001, and **** *p* < 0.0001; n = 4–8/group).

Complement proteins produced by renal tubular epithelial cells have been proposed as critical players in the development of renal fibrosis [32]. Gene expression analyses at three collection time points (1/3/7 days) showed dysregulations in the complement C3 mRNA expressions between the B6, CBA, and BalbC mouse kidneys (Figure 7a). In UUO kidneys on Day 1, *C3* expression increased significantly in CBA and BalbC but not in B6 UUO kidneys (Figure 7a). As compared to CBA and BalbC, B6 UUO kidneys depicted a delayed but progressive increase from Day 3 to 7, whereas CBA kidneys showed a 2-fold higher *C3* expression on Day 3 than B6 UUO kidneys. However, the *C3* mRNA expression in UUO kidneys reached equally high levels in all strains by Day 7 (Figure 7a).

Complement Factor H (CFH) is an inhibitor of C3 through the alternative pathway, so we wanted to analyze its time-dependent mRNA expression pattern in the different mouse strains. Interestingly, we only observed early, but statistically inignificant, *Cfh* transcription activity in B6 UUO kidneys on Day 1, which progressively rose by Day 3 and Day 7 (Figure 7b). We observed significantly delayed over-expression patterns of *Cfh* in CBA and BalbC UUO kidneys from Day 3 to Day 7 as compared to B6 UUO ones. On Day 7, the expression in BalbC UUO kidneys remained significantly lower than that in B6 UUO ones (Figure 7b).

In contrast to the mRNA expression pattern, the kidney C3 protein expression (Figure 7c) on Day 1 after UUO was not significantly elevated, but reached statistical significance on Day 3, when CBA UUO kidneys depicted the highest protein expression.

### 2.6. Renal C3 Over-Production Was Mainly Localized to Renal Tubules

**Figure 8:**
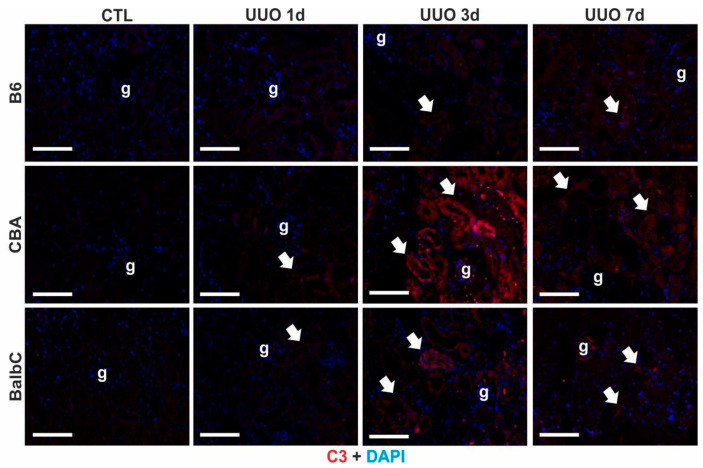
Immunofluorescent staining of complement C3 in mouse kidneys at 1/3/7 days after UUO. Control (CTL) and obstructed (UUO) kidneys of B6, CBA, and BalbC mice on Days 1, 3, and 7 were stained with C3 (red) and 4′,6-diamidino-2-phenylindole (DAPI) nuclear stain (blue). Immunoreactivity was localized to tubules (white arrows); glomeruli (g) were not stained. Magnification is 400×; the scale bar represents 50 μm.

Based on the evaluation of the gene and protein expressions in the UUO kidneys, we aimed to localize C3 in the kidney tissues with immunohistochemistry. On Day 1, despite the early and dramatic mRNA over-expression in CBA and BalbC kidneys, we could not detect significant C3 immunoreactivity compared to the contralateral controls in any UUO kidneys of all three strains. On Day 3, however, mild tubular C3 staining appeared in B6 UUO samples. Extensive C3 immunoreactivity was localized in the tubular epithelial cells of CBA and BalbC kidneys (Figure 8, see arrows), where CBA tubules exhibited the highest expression. By Day 7, the C3 immunostaining of the UUO kidneys decayed in all three strains, but was still visible in CBA UUO kidneys (Figure 8).

### 2.7. BalbC and CBA Kidneys Depicted Prominent Early Inflammation upon UUO

**Figure 9:**
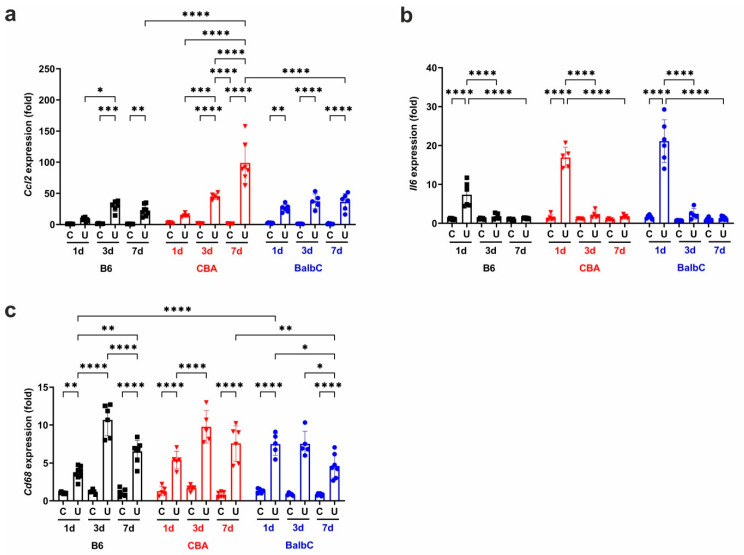
Strain- and time-dependent renal mRNA expressions of inflammatory markers after UUO. Relative fold change mRNA expressions of (**a**) CCL2 (*Ccl2*), (**b**) IL-6 (*Il6*), and (**c**) CD68 antigen (*Cd68*) in control contralateral (C) and obstructed (U) kidneys of B6, CBA, and BalbC mice were calculated against *18S* rRNA expression. Data were analyzed via two-way ANOVA followed by the Holm–Sidak’s multiple comparison test (* *p* < 0.05, ** *p* < 0.01, *** *p* < 0.001, and **** *p* < 0.0001; n = 5–8/group).

Ureter obstruction induced early (Day 1) and robust mRNA over-expression of the inflammatory marker CCl2 in BalbC mice (Figure 9a), but not in B6 or CBA mice. The expression of *Ccl2* increased progressively by Day 3 in all strains, culminating in CBA UUO kidneys having the highest expression on Day 7. In contrast, interleukin-6 (IL-6) mRNA (*Il6*) was induced only on Day 1 and reached the highest values in BalbC UUO kidneys. From Day 3, *Il6* expression showed a dramatic resolution in UUO kidneys in all strains, reaching levels similar to those measured in the controls (Figure 9b). The mRNA expression of the macrophage marker cluster of differentiation 68 (CD68) antigen (*Cd68*) increased early in parallel with *Il6*, reaching the highest values in BalbC UUO kidneys on Day 1 (Figure 9c). From Day 3, the *Cd68* levels remained elevated in UUO kidneys to a similar extent in all strains.

### 2.8. C3a Receptor Agonist In Vitro Induces an Inflammatory and Pro-Fibrotic Response in Primary Tubular Epithelial Cells of Mice

**Figure 10:**
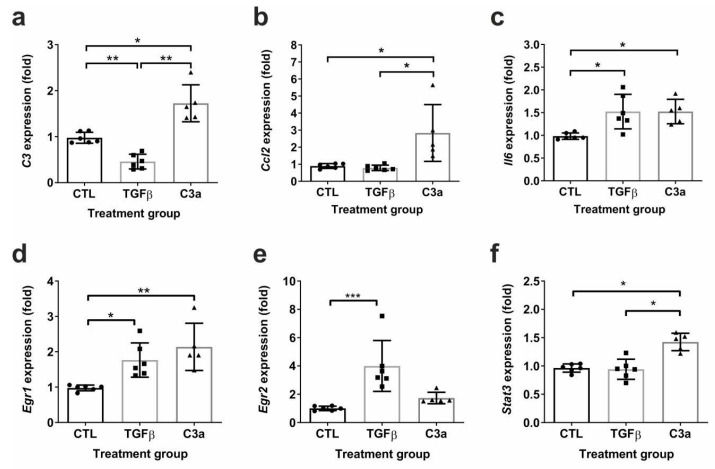
Inflammatory and transcription factor mRNA expression of PTECs upon TGFβ and C3a receptor agonist treatment. Relative fold change mRNA expressions of (**a**) C3 (*C3*), (**b**) CCL2 (*Ccl2*), (**c**) IL-6 (*Il6*), (**d**) EGR1 (*Egr1*), (**e**) EGR2 (*Egr2*), and (**f**) STAT3 (Stat3) in mouse PTECs after 24 h treatment with phosphate-buffered saline (PBS) (CTL), TGFβ, and C3a agonist. Expressions were calculated against *18S* rRNA expression, and data were analyzed via one-way ANOVA followed by the Holm–Sidak’s multiple comparison test (* *p* < 0.05; ** *p* < 0.01; and *** *p* < 0.001; n = 5–6/group).

Based on the in vivo results, we aimed to clarify how the renal production of C3 would influence the process of tubulointerstitial fibrosis via tubular epithelial cell effects compared with the pro-fibrotic TGFβ. The treatment of the primary tubular epithelial cells with a C3a receptor agonist (C3a) induced the transcription of *C3*, *Ccl2*, and *Il6* mRNA, while TGFβ treatment only upregulated the expression of *Il6* while suppressing *C3* (Figure 10a–c).

Both C3a and TGFβ treatments induced upregulation of the *Egr1* transcription factor (Figure 10d), but only TGFβ significantly upregulated *Egr2* in PTECs (Figure 10e). In contrast, *Stat3* expression was upregulated only by C3a, but not by TGFβ treatment (Figure 10f).

**Figure 11:**
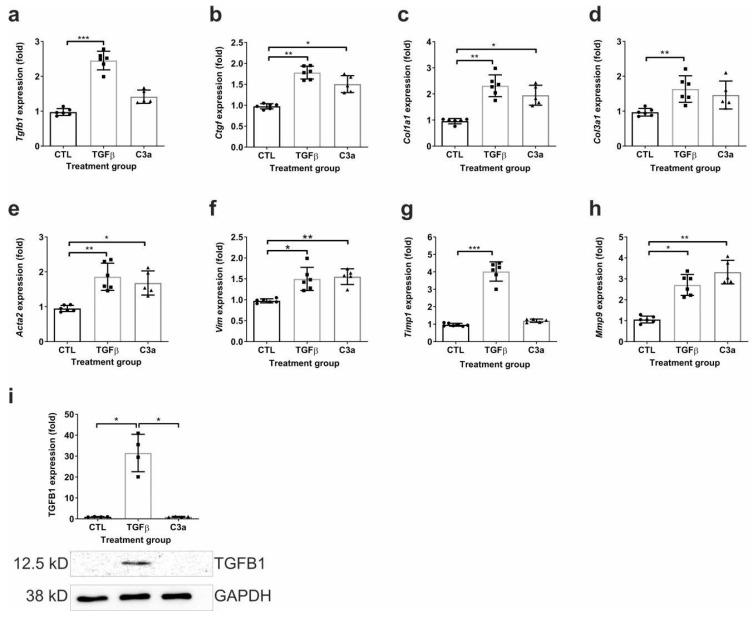
Fibrosis-related gene and protein expressions of PTECs upon TGFβ and C3a receptor agonist treatment. Relative fold change mRNA expressions of (**a**) TGFβ1 (*Tgfb1*), (**b**) CTGF (*Ctgf*), (**c**) type I collagen (*Col1a1*), (**d**) type III collagen (*Col3a1*), (**e**) α-SMA (*Acta2*), (**f**) vimentin (*Vim*), (**g**) TIMP-1 (*Timp1*), and (**h**) MMP-9 (*Mmp9*), and (**i**) protein expression of TGFβ1 (TGFB1) in mouse PTECs after 24 h treatment with PBS (CTL), TGFβ, and C3a agonist. Gene and protein expressions were calculated against 18S rRNA or tubulin expression. Data were analyzed via one-way ANOVA followed by the Holm–Sidak’s multiple comparison test (* *p* < 0.05; ** *p* < 0.01; and *** *p* < 0.001; n = 4–6/group).

The mRNA expressions of the fibrosis-related molecules TGF-β1 (*Tgfb1*) and TIMP-1 (*Timp1*) were markedly upregulated by TGFβ, but not by C3a treatment (Figure 11a,g), and TGF-β1 protein expression showed similar pattern (Figure 11i). However, *Ctgf*, *Col1a1*, and *Mmp9* levels were induced similarly by both the C3a and TGFβ treatments (Figure 11b,c,h), while Col3a1 responded to TGFβ treatment only (Figure 10d). Among the markers of EMT, the mRNA expressions of both α-SMA (*Acta2*) and vimentin (*Vim*) were significantly upregulated by both the C3a and TGFβ treatments (Figure 11e,f).

**Figure 12:**
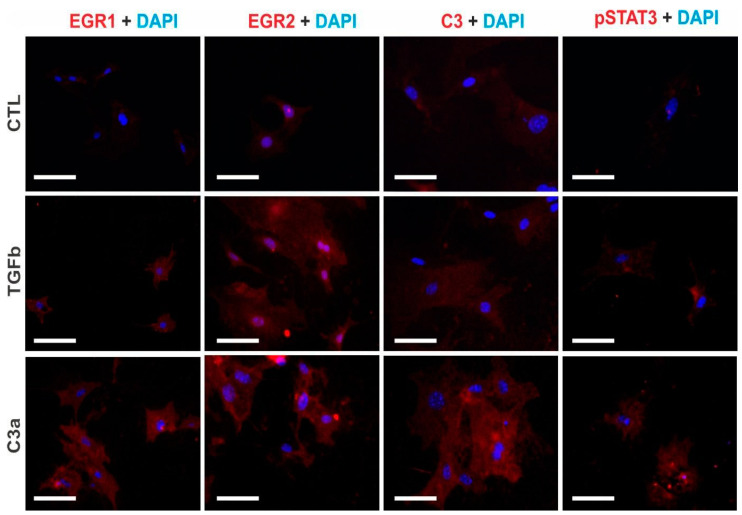
Immunocytochemistry of EGR1, EGR2, C3, and pSTAT3 after TGFβ and C3a treatments in PTECs. After 24 h of treatment of PTECs with PBS (CTL), TGFβ, or C3a, immunofluorescence images of protein expressions (in red) were assessed for EGR1, EGR2, C3, and Tyr705-phosphorylated STAT3 (pSTAT3). DAPI was used for nuclear staining (blue). Scale bars represent 25 μm at magnification 630×.

Immunocytochemistry revealed over-expressions of the EGR1, EGR2, and C3 proteins in the cytoplasm and STAT3 phosphorylation after the 24 h C3a agonist treatment (Figure 12), corroborating the gene expression results. TGFβ treatment induced EGR2 protein expression, nuclear translocation, and, to a lesser extent, EGR1 protein expression (Figure 12).

### 2.9. Renal C3 mRNA and Protein Expressions Are Upregulated in Human FSGS Kidneys

**Figure 13:**
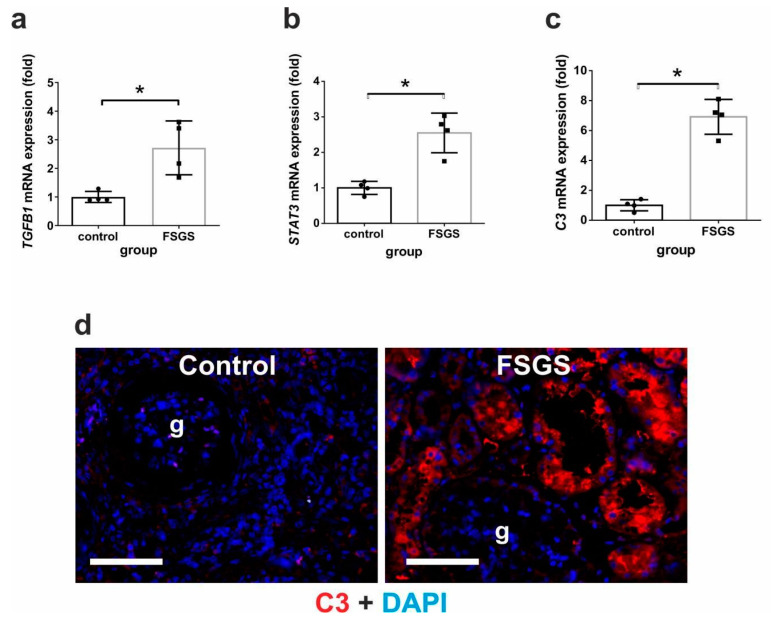
Analysis of human kidneys from FSGS patients and controls. Relative fold change mRNA expressions of (**a**) TGFβ1 (*TGFB1*), (**b**) *STAT3*, and (**c**) *C3*, and (**d**) immunohistochemistry for C3 in human biopsies of control and FSGS kidneys (red: C3, blue: DAPI for nuclear stain; g: glomerulus; scale bar represents 50 μm). Gene expressions were calculated against *18S* rRNA. Data were analyzed with the Mann–Whitney test (* *p* < 0.05; n = 4/group).

To test whether the fibrosis-associated upregulation of renal C3 is present in fibrotic human kidney disease, biopsies of kidneys with FSGS compared to healthy controls were analyzed. As expected, we observed significantly elevated TGFβ1 mRNA expression (*TGFB1*) in the FSGS biopsies (Figure 13a), accompanied by upregulated *STAT3* expression (Figure 13b). In addition, we observed a marked upregulation of renal *C3* at the mRNA (Figure 13c) and protein levels, localized mainly to tubular epithelium (Figure 13d). These data imply that the renal de novo production of C3 might also be involved in the pathogenesis of human kidney fibrosis.

## 3. Discussion

Here, we first demonstrate a strain-dependent variation in murine kidney fibrosis progression associated with de novo tubular epithelial C3 production. We further demonstrate that the activation of the C3a receptor exacerbates the epithelial-to-mesenchymal transition (EMT) and exerts strong inflammatory and pro-fibrotic tubular epithelial cell responses in vitro, comparable to TGFβ-induced effects but independent of the TGF-β pathway.

After UUO surgery, the increasing intra-tubular pressure proximal to the obstruction (ligation) site causes mechanical stress on the epithelium and dilates the tubules. This injury to the tubules precedes the development of interstitial fibrosis, supported by in vitro studies that demonstrated cortical fibroblast proliferation stimulated by paracrine signals in co-culture [33]. The prolonged mechanical tension from the ureteral obstruction leads to fibroblast proliferation and the secretion of pro-fibrotic stimuli, increasing ECM stiffness to counteract mechanical stress from the tubular system [34]. In our study, the early up-regulation of lipocalin-2 (*Lcn2*) (or NGAL) mRNA in the UUO kidneys also preceded any significant histological alterations at twenty-four hours, regardless of the mouse strain.

Damage to the tubular epithelial cells produces pro-fibrotic factors and transcription factors, exhibiting a paracrine effect on resident fibroblast/pericytes [35]. The essential involvement of the TGF-β pathway in the development of renal fibrosis has been well described in vitro and in vivo [36,37], leading to the accumulation of extracellular matrix (ECM) components, such as collagen I and collagen III. Several murine experiments have demonstrated that C57Bl/6J mice (B6), including transgenic or surgery models, are less susceptible to kidney fibrosis [8,38]. Also, we showed recently that the CBA mouse strain is prone to renal fibrosis induced by TGF-β, unilateral ureter obstruction, or renal mass reduction [8]. A study using the Adriamycin nephropathy model concluded that BalbC mice are more sensitive than B6 mice, and that locally synthesized complement proteins are involved in developing renal injury associated with glomerular damage and proteinuria [39]. In our studies, the gene expressions of collagens increased in parallel with TGFβ1 and CTGF as early as twenty-four hours after UUO in B6, CBA, and BalbC kidneys. However, according to previous reports, the B6 kidneys depicted a milder renal TGFβ1 upregulation and delayed fibrosis development than CBA and BalbC kidneys. CTGF acts as a transcription factor and a pro-fibrotic mediator downstream of TGF-β [40,41,42]. Our in vivo data indicate that *Ctgf* depended on *Tgfb1* expression in the UUO kidneys of all strains. However, the transcription of *Ctgf* but not *Tgfb1* was induced by the C3a receptor agonist in vitro, which also suggests a direct effect on CTGF, independent of TGFβ.

The dysregulation of ECM component production and degradation by matrix metalloproteinases (MMPs) and their tissue inhibitors (TIMPs) contributes to fibrosis development [43]. TIMP-1 might play a crucial role in kidney fibrosis [8]. Like our previous studies, UUO induced a very early upregulation of *Timp1* in all mouse strains, but to a significantly lesser extent in B6 kidneys. On the other hand, *Mmp2* expression was induced late in all strains, on Day 7, while *Mmp9* started to increase on Day 3, when histology first approved the presence of fibrosis. Interestingly, the C3a receptor agonist in vitro induced *Mmp9* but not *Timp1* expression, while TGFβ treatment activated both genes in PTECs.

Complement factors involve essential processes, including cell proliferation and survival [44] or tissue regeneration [45]. However, the over-activation of the complement system following an injury can drive sclerotic changes in tissue. C3 can be activated by the classical, alternative, or lectin pathways depending on the stimulus [45], but the renal activation pathway of C3 differs from that of other circulating cells. For instance, both interleukin-1 alpha (IL-1α) and interleukin-2 (IL-2) were reported to induce the local synthesis of C3 in vitro in human proximal tubular epithelial cells (hPTECs) [46], while tumor necrosis factor (TNFα) and interferon gamma (IFNγ) had no effect [31]. Serum proteins stimulated C3 production in hPTECs [47], and transferrin, but not albumin, also induced C3 production in hPTECs in a dose-dependent manner [29].

On the other hand, intracellular C3 was recently found to maintain homeostatic cellular processes, apoptosis, autophagy, and the regulation of inflammation [48]. Our experimental data from the C3 mRNA expressions and tubular C3 protein localization in the UUO kidneys indicate the de novo synthesis of complement C3 protein by the renal tubular epithelium, confirmed in our PTECs in vitro. The lower expression level of C3 in the renal-fibrosis-resistant B6 mice vs. renal-fibrosis-susceptible CBA and BalbC strains is in concordance with renal histology, suggesting that complement proteins synthesized within the kidney play a more profound role in the development of kidney fibrosis than the circulating complement factors. We further confirmed the possible pathogenic role of renal epithelial C3 production in human fibrotic kidney biopsies. It is speculated that all known branches of the complement cascade (classical, lectin, and alternative) are involved in the progression of renal disease [32].

Furthermore, C3a was reported to facilitate the production of inflammatory factors [49]. C3a was reported to induce STAT3, resulting in an inflammatory response and fibrosis in rodents [50], while C3aR deficiency in mice reduced tubulointerstitial inflammation and kidney fibrosis [22]. Previous reports are consistent with our observations, where the C3aR agonist treatment induced *Ccl2* and *Il6* transcription within twenty-four hours in vitro. These results implicate C3aR blockade as an anti-fibrotic treatment option. The seldom reports of C3aR antagonist treatment in Type II diabetic rats ameliorating diabetic nephropathy hold discrepancies as to the observed effect being due to the suppression of the TGF-β/Smad3 pathway [51], which is inconsistent with our results.

Recent studies have identified that the STAT3 transcription factor is implicated in CKD progression. In the murine subtotal nephrectomy (SNX) model, tubular STAT3 promoted the activation of interstitial fibroblasts by the induction of *Lcn2* and *Timp1* [52], and recent data suggest a direct activation by TGFβ [53]. In our present study, renal STAT3 remained active (phosphorylated) in UUO kidneys from Day 3 to Day 7, even after the resolution of *Il6* transcription by Day 7. In addition, our in vitro study showed that a C3aR agonist can directly induce tubular STAT3 transcription independent of TGFβ, thus contributing to the inflammatory and pro-fibrotic phenotype.

IL-6 is a pro-inflammatory mediator in fibrotic processes and acts as a transcriptional inducer of CTGF [54]. In our study, *Il6* was differentially expressed in all three mice strains at twenty-four hours, suggesting variability in the initial inflammatory response to injury. Due to the involvement of the IL-6/STAT3 pathway in renal fibrosis, the blockade of STAT3 phosphorylation is a promising therapeutic approach in the UUO model in vivo [55].

Myofibroblast activation and proliferation is a hallmark of fibrosis [56]. Thus, 5h3 epithelial-to-mesenchymal transition (EMT) has been perceived as one of the pathogenic contributors in kidney fibrosis development, characterized by a loss of epithelial markers and upregulating mesenchymal markers such as SMA or vimentin. Several studies have identified the EMT as a prominent in vitro feature of tubular epithelial cells [57,58]. In our experiments, renal SMA (*Acta2*) expression was similarly induced by UUO in all strains. Studies have demonstrated that C3a/C3aR may initiate the EMT, tubulointerstitial inflammation, and fibrosis in proximal tubular epithelial cells mediated by the TGF-β1/CTGF signaling pathway [59,60]. In our studies, however, we observed that the C3aR agonist exerted similar effects as TGFβ, an upregulation of *Acta2* and vimentin without the induction of TGFβ signaling in PTECs. This suggests that C3 might directly induce the EMT on tubular epithelial cells in vitro, independent of TGFβ.

The transcription factors early growth response protein-1 and -2 (EGR1 and EGR2) can stimulate collagen synthesis and myofibroblast differentiation and are associated with TGFβ and fibrosis progression [8,53]. Both *Egr1* and *Egr2* were upregulated early after UUO in all strains, but the *Egr2* response was delayed in B6 kidneys, corroborating our histology findings and previous work [8]. In the present study, the early renal expression of *Egr1* seemed more closely related to C3 than *Tgfb1*, suggesting an alternative pathway for *Egr1* induction in vivo after UUO surgery. Based on our in vitro results, it is very likely that locally produced C3 after kidney injury might directly induce EGR1 in tubular epithelial cells via C3aR stimulation.

We also observed the early renal over-expression of the transcription factor CREB5 in all three mice strains on Day 3 and Day 7 after UUO. Previous studies have also reported upregulated CREB5 in the renal proximal tubules [61] and kidneys of mice subjected to UUO [62]. In addition, CREB5 has been associated with the mesenchymal cell signature [63] and has been reported to be upregulated in pre-activated fibroblasts along with C3 [63]. In B-lymphocytes, C3/C3a was observed to induce the transcription of CREB5 [48], although this pathway needs to be verified in renal cells. In our study, *Creb5* and *C3* exhibited high expression levels in BalbC UUO kidneys at twenty-four hours. On the other hand, the delayed renal *Creb5* response to UUO in the B6 kidneys might also contribute to the milder fibrosis in this mouse strain.

Focal segmental glomerulosclerosis (FSGS) is a histopathologic lesion with multiple causes and pathogenic mechanisms, characterized by the gradual loss of nephrons and hemodynamic stress leading to proteinuria. FSGS is categorized into primary (or idiopathic) and secondary FSGS, referring to their etiologies. Secondary FSGS is caused by known factors, such as nephron loss, infection, or medication. The causes for primary FSGS are virtually unknown and are manifested by a primary loss of podocytes in the glomerulus, followed by progressive glomerular scarring and tubulointerstitial fibrosis. A genetic predisposition to FSGS has been proposed. The presence or absence of nephrotic-range proteinuria in patients influences the direction of their therapy, with a major difference in the use of immunosuppressive medications when addressing populations of European ancestry [64].

Regarding current medical practices, all FSGS patients are treated with either ACEi (angiotensin-converting-enzyme inhibitor) or ARB (angiotensin receptor blocker). Among our kidney biopsy patients, three out of four were taking either ARB or ACEi at the time of biopsy, but only one patient had Grade I hypertension despite the medication. Considering that statistically more patients with diabetes develop primary FSGS, they would also take accompanying diabetic medications to control comorbidities. Furthermore, increased renal complement C3 has been associated with an increased blood pressure both in experimental models and humans [65]. A meta-analysis of randomized control trials failed to establish the benefits of ACEi/ARB monotherapy in the heterogenic population, which might indicate the need for additional supporting therapies to be explored [66]. In addition, there is uncertainty about using immunosuppressive therapies in patients with primary FSGS [67]. Among the investigated kidney biopsies, 50% of the patients had steroid-resistant nephrotic syndrome, and they received immunosuppressive treatment. The simultaneous inhibition of G-protein-coupled receptors has been suggested as a therapy alternative for adult FSGS patients (ANGII and CCR2), and a Phase 3 randomized trial (NCT05183646) is currently recruiting. C3aR is also a G-protein-coupled receptor and could be a potential candidate for simultaneous inhibition along with ARBs targeting inflammatory pathways [20]. In addition, C3, C5, and C5aR inhibitors have shown efficacy for FSGS treatment in pre-clinical data [68].

### Study Limitations and Translational Importance

Our study has some limitations. The UUO mouse model only partly resembles human tubulointerstitial fibrosis in CKD, as similar complete ureter obstruction is only seen in pediatric patients with developmental anomalies. Still, the aggressive UUO model initiates rapid cellular responses and allows for a better (and significantly faster) observation of molecular mechanisms during progression driven by genetic differences, as compared to, for instance, renal ablation models. This feature of UUO allowed us to investigate very early molecular changes (within 24 h) when renal histology remained normal. The identification of these early molecular mechanisms might contribute to earlier diagnoses and interventions in human CKD.

Another limitation of our study is that due to its small sample size, the heterogeneous age distribution, and the lack of genetic testing, we could not clearly identify the etiology of FSGS in most of the cases; therefore, our rather small patient population presumably might contain both primary and secondary forms. The lack of some clinical parameters, serum C3 level measurements, or CKD staging at time of biopsy further limit the interpretation of the FSGS results, as we were not able to correlate gene expression data with CKD progression markers.

However, the STAT3 and C3 mRNA expressions were elevated to a similar extent in all FSGS samples, regardless of the underlying disease, emphasizing that the renal complement system and STAT3 activation might play important roles in FSGS pathogenesis. Further clinical research needs to test the efficacy of emerging medications that inhibit STAT3 and/or C3aR activation. These new interventions might represent a promising future therapy for FSGS patients.

## 4. Materials and Methods

### 4.1. Mice

Six-week-old C57Bl/6J (B6), CBA/J (CBA), and BALB/cJ (BalbC) male mice (Jackson Laboratories, Bar Harbor, ME, USA) weighing 19–22 g were housed under standard specific pathogen-free (SPF) conditions (Semmelweis University NET GMO facility, Budapest, Hungary), with a 10/14 h light/dark cycle at 22 °C and 60% relative humidity. They were provided with food and drink ad libitum. The animals were sacrificed under deep diethyl-ether narcosis. The Semmelweis University Institutional Ethical Committee approved all animal experiments (PE/EA/948-4/2018).

### 4.2. Unilateral Ureteral Obstruction (UUO)

The animals were anesthetized with an intraperitoneal injection of ketamine/xylazine (100 mg/kg body weight ketamine and 10 mg/kg xylazine). To perform UUO, the left kidney and ureter were exposed after median laparotomy under sterile conditions; the ureter was ligated at approximately 0.5 cm (proximal) and 1.0 cm (distal) below the kidney with 4.0 silk sutures and cut between ligatures. Volume depletion was prevented by the administration of 0.1 mL of saline into the peritoneal cavity. The midline incision was closed, and the mice were returned to their cages and allowed free access to food and water. Twenty-four hours (Day 1), three days (Day 3), and seven days (Day 7) post-surgery, the mice were euthanized. The mice were perfused via the heart’s left ventricle for 20 min with ice-cold saline (containing 0.1% procaine hydrochloride (Merck, Budapest, Hungary)). The UUO and contralateral (control) kidneys were collected for analysis.

### 4.3. Cell Culture

Primary tubular epithelial cells (PTECs) were isolated from four-week-old male C57B1/6J mice and characterized as previously described [69]. For treatment, the cells were seeded at a density of 1,000,000 cells/well in gelatin-coated six-well plates. Twenty-four hours before treatment, the cells were maintained in serum-free DMEM-F12 medium and then incubated with 100 nM C3a receptor agonist peptide ((Trp63, Trp64)-C3a (63–77), Bachem, VWR, Budapest, Hungary) or 10 ng/mL human recombinant TGF-β1 (Merck) for 24 h.

### 4.4. Human Kidney Biopsy Samples

Frozen kidney cortex core biopsy samples, histologically diagnosed with focal segmental glomerulosclerosis (FSGS), were obtained from the 1st and 2nd Department of Pathology, Semmelweis University. Two of the patients developed FSGS due to hypertension, and the two young patients had steroid-resistant nephrotic syndrome as an underlying disease (demographic data are presented in Supplementary Table S1). The use of frozen kidney specimens was ethically approved by the Semmelweis University Ethical Board (TUKEB 228/2014). Normal kidney cortex samples were obtained from healthy areas of kidneys nephrectomized due to renal cell carcinoma. RNA was extracted and qPCR studies were performed as described below.

### 4.5. Renal Histology and Immunohistochemistry

Perfused kidneys were removed and fixed in 4% buffered formalin or snap-frozen in liquid nitrogen. Then, 4 µm sections were cut from the paraffin-embedded specimens and subjected to Masson’s Trichrome staining or further processed for immunohistochemistry and stained with rabbit monoclonal anti-C3 (1:200, Abcam, Cambridge, UK). Antigen retrieval was performed with 0.1 M citrate buffer, pH 6.0, in a water bath at 95 °C for 20 min. Sections were blocked with 5% goat serum in PBS-Tween for 30 min, then incubated with secondary antibody (1:200, goat anti-rabbit-Alexa-594, Jackson Immunoresearch, West Grove, PA, USA) for 2 h in the dark. The slides were then covered with coverslips on VectaShield mounting medium containing DAPI for nuclear staining (Vector Laboratories, Newark, CA, USA) and analyzed under a fluorescent microscope.

The extent of tubulointerstitial damage was evaluated on trichrome-stained sections as previously described [70], in every field of view, at ×400 magnification. The scores for each alteration were as follows: for tubular dilatation, 0 = none or 1 = dilated tubuli present; for tubular atrophy, 0 = none or 1 = signs of atrophy and desquamation of cells; for tubular hyaline, 0 = none or 1 = hyaline present; for interstitial fibrosis, 0 = none, 1 = mild, or 2 = severe; and for mononuclear cell infiltration, 0 = none, 1 = mild, or 2 = severe. Tubulointerstitial damage scores are expressed as the arithmetic mean of the total scores in all fields. All samples were evaluated in a blinded manner.

### 4.6. Quantitative RT-PCR

According to the manufacturer’s protocol, kidney samples (50 mg) were homogenized, and the total RNA was extracted using TRIzol (Invitrogen, Thermo, Waltham, MA, USA). The RNA concentration was determined photometrically. The purity of the isolated RNA was verified by the A230/A260 and the A260/A280 absorbance ratios (cut-off values for both were 1.6) calculated by a Nanodrop 2000 Spectrophotometer (Thermo, USA). The RNA integrity was verified by electrophoretic separation on an ethidium-bromide stained 1.5% agarose gel and calculating the 28S to 18S rRNA ratio. Samples with signs of partial degradation (smeared RNA lacking sharp rRNA bands or a 28S:18S ratio lesss than 2:1) were excluded from further studies. Reverse transcription was performed with the High-Capacity cDNA Reverse Transcription kit from Applied Biosystems (Applied Biosystems/Life Technologies, Carlsbad, CA, USA) using 2 μg RNA and random primers in a final volume of 10. All PCR reactions were performed on a Bio-Rad CFX thermal cycler (Bio-Rad Hungary, Budapest, Hungary) using the Power SYBR Green PCR Master Mix (Applied Biosystems). Melting and standard curve analyses confirmed the PCR reaction’s specificity and efficiency. Every sample was quantified in duplicate and normalized to 18S ribosomal RNA expression. Mean values are presented as the fold expression relative to a B6 control sample using the formula 2-ΔΔCt. All primers were purchased from Merck (Darmstadt, Germany). The primer sequences are shown in Table 1.

### 4.7. Immunoblot

Kidney samples (approximately 20 mg) were homogenized in RIPA lysis buffer containing a complete protease inhibitor cocktail (Roche, Mannheim, Germany) and homogenized. Cells were incubated in the same ice-cold lysis buffer for 20 min on ice. The protein concentration was determined by the BCA Assay (Thermo Scientific, Waltham, MA, USA). The samples were mixed with 2× Laemmli buffer and boiled at 95 °C for 5 min. Equal amounts of protein (25 µg) were separated on 12% SDS-polyacrylamide gel on a Mini Protean Tetra Cell system (Bio-Rad), transferred to nitrocellulose membranes, and blocked with 5% skim milk in Tris-buffered saline (TBS) containing 0.1% Tween-20. The membranes were incubated overnight at 4 °C with the following antibodies: rabbit polyclonal C3 (1:2000, Abcam, UK) and mouse monoclonal tubulin (1:10,000, Sigma-Aldrich, St. Louis, MO, USA). The membranes were washed and incubated with peroxidase-conjugated secondary antibody (anti-mouse IgG, 1:10,000, or anti-rabbit IgG, 1:2000, Cell Signaling, Danvers, MA, USA). Blots were visualized by an ECL detection kit (Thermo Scientific, USA).

### 4.8. Immunofluorescence

A total of 30,000 cells were grown on gelatin-coated glass coverslips. The cells were fixed in methanol (−20 °C) for 15 min on ice, permeabilized, and blocked using 0.25% Triton-X and 2% donkey serum in phosphate-buffered saline (PBS). The cells were incubated overnight at 4 °C with the following primary antibodies: rabbit anti-C3a (1:300, Abcam, UK) and rabbit anti-TGFβ (1:200, Cell Signaling, USA) in diluent (2% goat serum in TBS-T). The next day, the cells were incubated with a secondary antibody (1:200, anti-rabbit-Alexa-594, Jackson Immunoresearch, West Grove, PA, USA) at room temperature for 1 h in the dark. Coverslips were transferred onto microscope slides and mounted with VectaShield containing DAPI.

### 4.9. Statistics

The required sample sizes for each group were estimated after conducting a pilot 3-day UUO experiment with 3 mice from each strain. Based on the pilot study results, the required minimum sample size was estimated using Mead’s formula and factorial analysis (90% statistical power and 5% statistical error, min. 50% mean deviation and 20% standard deviation) [71]. All statistical analyses were performed with GraphPad Prism 8 (GraphPad Software, La Jolla, CA, USA). Outliers were identified by using the GraphPad Prism outlier identifier function and were excluded from further analysis. Experimental data were tested for normal distribution with the Kolmogorov–Smirnov test and are presented as mean ± SD. Data were further analyzed with two-way ANOVA and Holm–Sidak’s multiple comparison test, or the Mann–Whitney U-test. The level of significance was set to *p* < 0.05.

## 5. Conclusions

**Figure 14:**
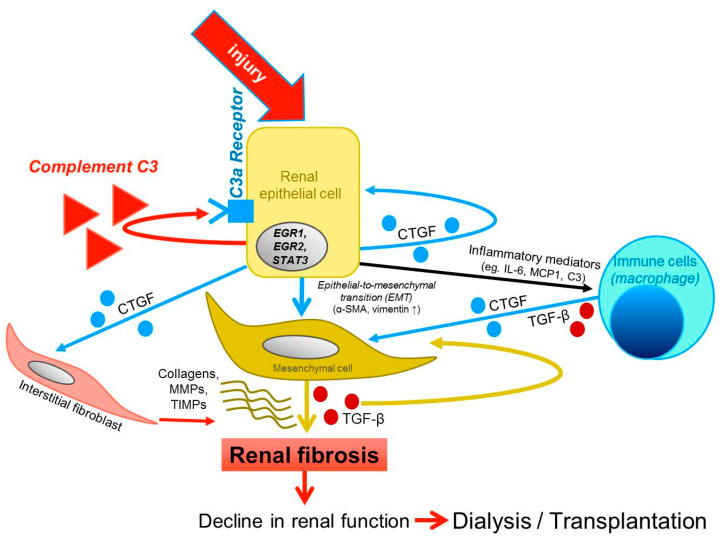
Schematic representation of how locally produced C3 might be involved in tubular epithelial cell injury and kidney fibrosis. Injured tubular cells release CTGF, inflammatory mediators, and C3 while undergoing EMT. The released CTGF exert its pro-fibrotic effects, while C3 activates its C3aR receptor in an autocrine/paracrine way, enhancing EMT and the pro-fibrotic program. Activated macrophages release CTGF and TGFβ that further promotes EMT. These self-propagating signals culminate in kidney fibrosis. Fibrosis reduces kidney function; thus the patient will need dialysis or kidney transplantation.

Local renal C3 production signals inflammation and repair. Paracrine and autocrine effects promote fibroblast differentiation, the accumulation of ECM components, and immune cell stimulation. A balance between mechanical and chemical stimuli will decide the final fate of the scarring tissue (Figure 14).

Our study showed that the local production of complement C3 in the kidney tubular epithelium is associated with strain-dependent fibrosis progression in mice. We first demonstrated that the C3aR stimulation of primary tubular epithelial cells exerts strong pro-fibrotic cellular responses, independent of the TGF-β pathway. Our studies implicate that local C3aR antagonist treatment could be a novel approach to dampen tubulointerstitial fibrosis in chronic kidney disease.

## Figures and Tables

**Table 1:** Murine and human primer sequences (5′-3′) used in qPCR analysis.

Gene Symbol	Forward Primer	Reverse Primer
Mouse primers		
*18S*	TGGTTGCAAAGCTGAAACTTAAAG	AGTCAAATTAAGCCGCAGGC
*Acta2*	ACATAGCTGGAGCAGCGTCT	CCCACCCAGAGTGGAGAA
*C3*	TCCTTCACTATGGGACCAGC	TGGGAGTAATGATGGAATACATGG
*Ccl2*	CACTCACCTGCTGCTACTCA	GCTTGGTGACAAAAACTACAGC
*Cd68*	GACCGCTTATAGCCCAAGGA	TCATCGTGAAGGATGGCAGG
*CfH*	CCGTATCAAGACATGTTCAG	GAAGGCAAGTTATTGATCCTG
*Col1a1*	CATAAAGGGTCATCGTGGCT	TTGAGTCCGTCTTTGCCAG
*Col3a1*	TGGAAAAGATGGAACAAGTGG	CCAGACTTTTCACCTCCAAC
*Creb5*	GCTCACCCAGACAAACATGC	CTGCATGGCTGTTATCGGAC
*Ctgf*	CCCGAGTTACCAATGACAATAC	CTTAGCCCTGTATGTCTTCAC
*Egr1*	TTCAATCCTCAAGGGGAGCC	TAACTCGTCTCCACCATCGC
*Egr2*	TGACCAGATGAACGGAGTGG	ACTCGGATACGGGAGATCCA
*Il6*	TCCTCTCTGCAAGAGACTTCC	TTGTGAAGTAGGGAAGGCCG
*Lcn2*	ACGTCACTTCCATCCTCGTC	CCTGGAGCTTGGAACGAATG
*Mmp2*	GGACAAGAACCAGATCACATAC	CGTCGCTCCATACTTTTAAGG
*Mmp9*	TGGATAAGGAGTTCTCTGGTG	CCACCTTGTTCACCTCATTTT
*Stat3*	CGACATTCCCAAGGAGGAGG	ACTTGGTCTTCAGGTACGGG
*Tgfb1*	CACCATCCATGACATGAACC	TCATGTTGGACAACTGCTCC
*Timp1*	CACGGGCCGCCTAAGGAACG	TCCGTGGCAGGCAAGGAAAGT
*Vim*	CAGAACATGAAGGAAGAGATG	TCCAGGTTAGTTTCTCTCAG
Human primers		
*18S*	ATCGGGGATTGCAATTATTC	CTCACTAAACCATCCAATCG
*C3*	GAACTGCCTTTGTCATCTTC	CAGACACGTACAAAGACTTC
*TGFB1*	GGAAATTGAGGGCTTTCGCC	CCGGTAGTGAACCCGTTGAT
*STAT3*	GAAACAGTTGGGACCCCTGA	AGGTACCGTGTGTCAAGCTG

## Data Availability

Experimental data are available upon reasonable request to the corresponding author.

## Supplementary Materials

The following supporting information can be downloaded at: https://www.mdpi.com/article/10.3390/ijms252312551/s1.
