# Supplementary material for: Renal Epithelial Complement C3 Expression Affects Kidney Fibrosis Progression
Source: Int J Mol Sci. 2024 Nov 22;25(23):12551. doi: 10.3390/ijms252312551 (PMC11640822; doi:10.3390/ijms252312551)
Supplement: Supplementary file 1 [file ijms-25-12551-s001.zip › ijms-3251424-supplementary.pdf]

**Title:**

# Renal epithelial complement C3 expression affects kidney fibrosis progression

Authors:

Ganna Stepanova [1], Anna Manzéger [1,2], Miklós M. Mózes [1,2] and Gábor Kökény [1,2,]*

[1] Institute of Translational Medicine, Semmelweis University, 1089 Budapest, Nagyvárad tér 4, Hungary;

[2] International Nephrology Research and Training Center, Semmelweis University, 1089 Budapest, Nagyvárad tér 4, Hungary

*Correspondence: kokeny.gabor@semmelweis.hu

## Supplementary information:

Supplementary Table S1.

**Supplementary Table S1.**

| Age | Gender | Underlying disease | Histology diagnosis | Serum creatinine (µmol/L) | Serum urea (mmol/L) | Serum albumin (g/L) | eGFR (ml/min) | Urine protein (mg/L) | Blood pressure (mmHg) | Medication (at time of biopsy) |
|---|---|---|---|---|---|---|---|---|---|---|
| 13 | female | Steroid resistant nephrotic syndrome | FSGS | 168 | 9.5 | 19 | N/A | N/A | N/A | ACEi (Enalapril), Furosemide, Methylprednisolone, Rosuvastatin |
| 14 | male | Steroid resistant nephrotic syndrome | FSGS | 41 | 4.3 | 31 | N/A | 412 | 122/67 | ARB (Losartan), Cyclosporine, Spironolactone |
| 29 | male | Hypertension, nephritic syndrome | FSGS | 268 | 15.3 | N/A | 24.6 | 1970 | 148/96 | ACEi (Ramipril), Thiazide |
| 67 | male | Hypertension, nephrotic syndrome | FSGS | 134 | 13.4 | 34 | 46.2 | N/A | 130/80 | No treatment. |

**Legend**. Demographic data of patients diagnosed with FSGS based on kidney biopsy, with basic laboratory parameters at the time of biopsy. (ARB: angiotensin receptor blocker; ACEi: angiotensin convertase inhibitor; N/A: not available).
